# The prognostic value of red cell distribution width-to-albumin ratio for 28-day mortality in sepsis patients: a multicenter analysis based on the eICU Collaborative Research Database

**DOI:** 10.3389/fmed.2026.1816709

**Published:** 2026-05-28

**Authors:** Mao Ye, Suqi Lv, Yuewei Li, He Huang, Dingchao Lv, Xiaolan Wang

**Affiliations:** 1 Changzhi Medical College, Changzhi, China; 2 Changzhi People's Hospital, Changzhi, China

**Keywords:** 28-day mortality, eICU Collaborative Research Database (eICU-CRD), red blood cell distribution width-to-albumin ratio (RAR), retrospective cohort study, sepsis

## Abstract

**Background:**

Sepsis remains one of the leading causes of death in intensive care units worldwide. The red blood cell distribution width-to-albumin ratio (RAR) is calculated by dividing the red blood cell distribution width (%) by serum albumin (g/dl); this indicator combines information on inflammation and nutritional status and is closely associated with the prognosis of critically ill patients. This study aimed to explore the association between RAR and 28-day all-cause mortality in adult patients with sepsis.

**Methods:**

This retrospective, multicentre cohort study extracted 13,888 adult patients meeting sepsis criteria from the eICU CRD database. Primary outcomes comprised 28-day ICU all-cause mortality and in-hospital all-cause mortality. Cox proportional hazards models assessed the independent association between RAR and mortality. Subgroup analyses and restricted cubic spline validation confirmed the consistency and nonlinear nature of this association.

**Results:**

A total of 13,888 patients with sepsis were included and divided into low-RAR and high-RAR groups based on the median RAR value (5.9). The 28-day mortality rate in the high-RAR group was significantly higher than that in the low-RAR group (23.0% vs 9.9%, *P* < 0.001). After multivariable adjustment, high RAR was independently associated with increased mortality (HR 1.50, 95% CI 1.36–1.64, *P* < 0.001). As a continuous variable, each unit increase in RAR was associated with an 8% higher risk of mortality (HR 1.08, 95% CI 1.07–1.10, *P* < 0.001). Subgroup analyses confirmed consistency across age, sex, and comorbidity stratifications. A non-linear dose-response relationship was observed, with mortality risk increasing progressively with higher RAR levels.

**Conclusion:**

Elevated RAR levels at admission are independently associated with increased 28-day mortality in patients with sepsis. As an easily accessible and low-cost indicator, RAR may prove to be a useful risk stratification tool in clinical practice; however, its clinical value in resource-limited settings still needs to be further validated through prospective validation and cost-effectiveness studies.

## 1 Introduction

Sepsis, a life-threatening condition characterized by dysregulated host response to infection, remains a formidable global health challenge and the primary cause of mortality in intensive care units (ICUs) (1–3). Despite advances in early recognition and management, its associated morbidity and mortality remain unacceptably high, imposing a substantial burden on healthcare systems worldwide (4). Short-term mortality, particularly within 28 days, serves as a critical endpoint for assessing disease severity and the efficacy of clinical interventions for sepsis, reflecting acute organ dysfunction and host response (5). Consequently, identifying reliable, readily accessible prognostic biomarkers is paramount for optimizing early risk stratification and individualized treatment strategies (6).

The quest for reliable prognostic indicators has driven exploration of various hematological and biochemical parameters. Among these, red cell distribution width (RDW)—a measure of red blood cell size variability—and serum albumin—a key marker of nutritional status and inflammation—have garnered independent attention. Elevated RDW correlates with increased inflammation, oxidative stress, and impaired erythropoiesis, and is associated with poorer outcomes in sepsis and other critical illnesses (7, 8). Conversely, hypoalbuminaemia reflects systemic inflammation, capillary leakage, and malnutrition, serving as a strong predictor of poor outcomes in septic patients (9–11). The ratio of RDW to albumin (RAR) integrates these two pathophysiological pathways, potentially offering a more comprehensive reflection of the combined effects of inflammatory dysregulation and nutritional impairment—hallmarks of septic pathophysiology (9, 11, 12).

In recent years, several studies based on large intensive care unit databases have preliminarily explored the prognostic value of RAR in sepsis. In the MIMIC-IV database, Xu et al. (9) included 14,639 adult patients with sepsis across all age groups and found that RAR was significantly associated with mortality at 28 days, 90 days and during hospitalization. Building on this, several studies have further focused on specific subgroups of sepsis patients: Gu et al. (10) demonstrated in patients with sepsis and concomitant atrial fibrillation (*n* = 3,042) that elevated RAR was associated with increased in-hospital mortality; Yao et al. (13) reported in patients with sepsis-associated delirium (*n* = 4,021) that a RAR ≥5.85 was independently associated with 30-day mortality; Wu et al. (2026) found a non-linear relationship between RAR and 30- to 365-day mortality (with an inflection point at 5.04) in patients with sepsis-associated acute respiratory distress syndrome (ARDS) (*n* = 6,042) (14). In the eICU-CRD multicentre database, two studies to date have focused on the elderly population: Hu and Qian (15) analyzed 17,321 elderly patients with sepsis and found a potential non-linear association between RAR and 28-day mortality, but did not formally validate this using methods such as restricted cubic splines (RCS); An et al. included 5,976 elderly (≥60 years) sepsis patients and reported a linear relationship using a generalized additive model (16).

Consequently, this study utilized the eICU CRD multicentre database to enroll adult patients with sepsis, with the aim of assessing the prognostic value of the RAR index for 28-day mortality (including in-ICU mortality and in-hospital mortality) in this broad population. Furthermore, the added value of this study is reflected in three aspects (Supplementary Table S1): (1) Unlike previous eICU-based studies that focused solely on elderly patients, we included adult sepsis patients of all ages, not limited to elderly or subgroups with comorbidities, thereby providing a more generalisable assessment; (2) This study is the first to utilize a threshold-based restricted cubic spline (RCS) method to systematically validate the non-linear dose-response relationship between RAR and 28-day mortality within the eICU-CRD multicentre database; (3) We formally validated consistency across key subgroups through interaction tests, a step previously absent in eICU-based analyses.

## 2 Materials and methods

### 2.1 Data source and ethics

This retrospective cohort study utilized data from the eICU Collaborative Research Database (eICU-CRD), a multicentre intensive care database containing de-identified clinical records from over 200,000 intensive care unit admissions across the United States (17). The establishment of this database was approved by the Institutional Review Boards of the Massachusetts Institute of Technology (MIT) and participating hospitals. Informed consent was waived due to the retrospective and anonymous nature of the data. Access to the database requires validation and authentication through a data usage agreement with the PhysioNet Review Board. Compliance with the Safe Harbor standards has been demonstrated (HIPAA certification number: 73425064). This study adheres to the ethical principles of the Declaration of Helsinki.

### 2.2 Study population

**Figure 1:**
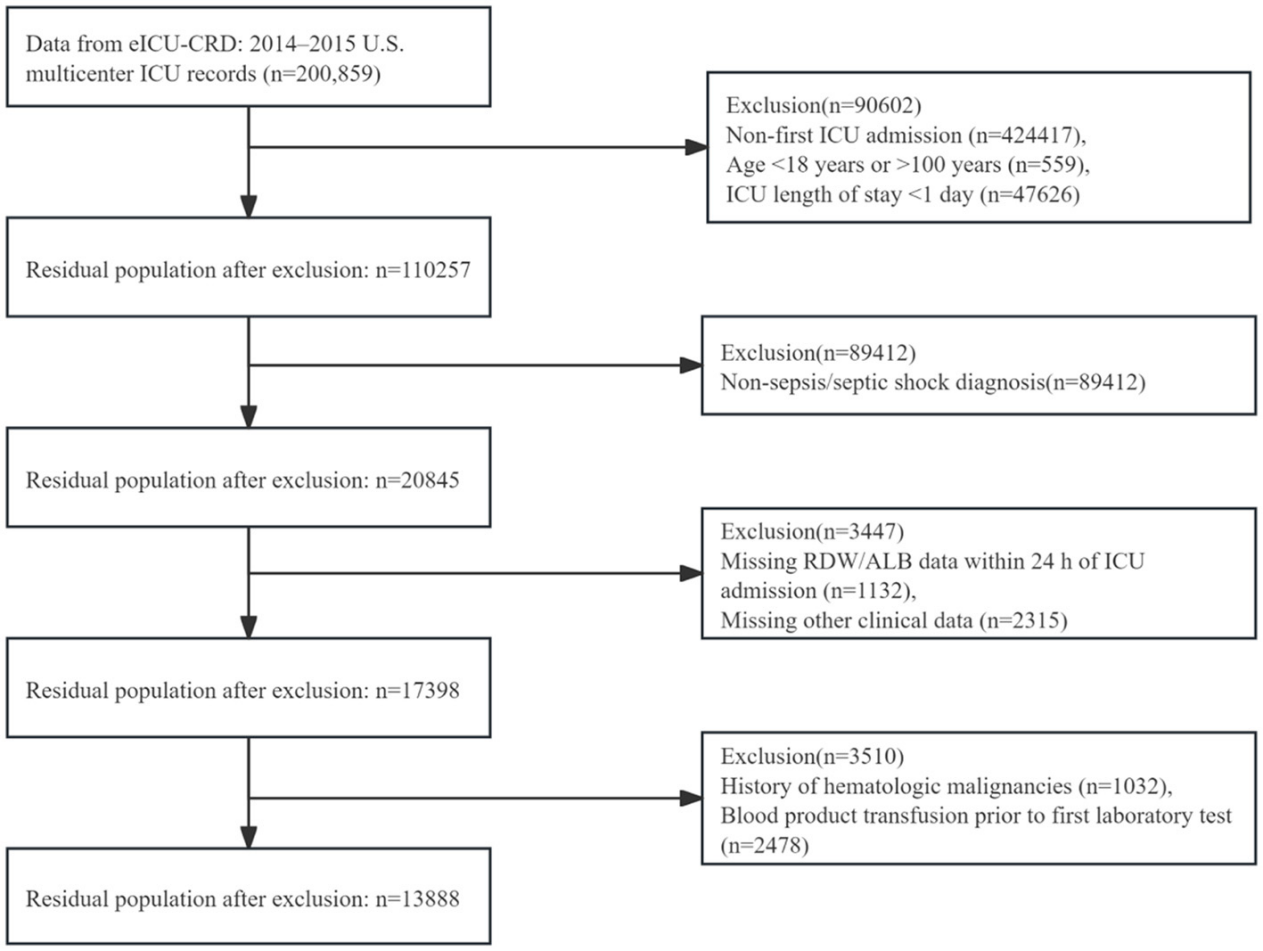
Flow chart of participant selection in the study.

The identification of sepsis follows the Sepsis-3 guidelines (18). Patients were diagnosed with sepsis on admission to the ICU if they met both of the following criteria: (1) the presence of a suspected or confirmed infection, documented in the admission record using pre-validated ICD-9-CM codes (0389, 99590–99592, 78552); and (2) a Sequential Organ Failure Assessment (SOFA) score increase of ≥2 points from baseline, calculated using the first available laboratory and clinical data within 24 h of ICU admission. For patients without a baseline SOFA score, a SOFA score of ≥2 at admission is considered an indicator of sepsis, consistent with the results of the previous eICU-CRD validation study. Exclusion criteria were: (1) The length of stay in the ICU was ≤ 24 h; this included 1,847 patients with sepsis, of whom 182 died, giving a mortality rate of 9.85% (182/1,847); (2) missing red cell distribution width (RDW) or serum albumin (ALB) data within 24 h of admission; (3) History of malignant hematological disease or previous bone marrow transplant; Related ICD-9-CM codes (20400, 20401, 20410, 20411, 20500, 20501, 20510, 20511, 99685); (4) patients receiving blood product transfusion prior to initial laboratory sampling. In the eICU database, transfusion events are recorded as a time offset (in minutes) relative to the time of admission to the ICU (drugstartoffset). Laboratory sample collection times are recorded using the same offset system. If the drugstartoffset value for the first transfusion is less than or equal to the offset value for the first RDW/albumin test sample collection time plus 30 min, the patient is excluded. A total of 13,888 eligible patients were ultimately included in the analysis (Figure 1).

### 2.3 Data collection and variable definitions

Demographic data, comorbidities, vital signs, laboratory parameters and disease severity scores (e.g. APACHE IV, SOFA, GCS) were extracted from the database. The primary exposure variable was the red blood cell distribution width-to-albumin ratio (RAR), calculated as RDW (%)/ALB (g/dl), using the first available measurement obtained within 24 h of the patient's admission to the ICU. In the primary categorical analysis, patients were divided into two groups based on the median RAR (RAR < 5.9 and ≥ 5.9) for analysis. Sensitivity analyses were performed using tertiles and quintiles. Quartile grouping was used solely for comparison with previous studies: Q1 (< 4.78), Q2 (4.78– < 5.85), Q3 (5.85– < 7.41) and Q4 (≥7.41) (19).

The primary outcome was the 28-day all-cause mortality rate following admission to the ICU, encompassing both in-hospital mortality and ICU mortality. Comorbidities such as hypertension, diabetes mellitus, chronic kidney disease, heart failure, and chronic obstructive pulmonary disease (COPD) were defined according to ICD-9 coding and clinical literature.

### 2.4 Statistical analysis

For normally distributed data, continuous variables are expressed as mean ± standard deviation (SD). For non-normally distributed data, continuous variables are presented as median (interquartile range, IQR). Categorical variables are reported as frequency (percentage) (19). Differences between the low RAR group and the high RAR group were compared using Student's *t*-test, Mann-Whitney U test, or chi-square test. A multivariate Cox proportional hazards regression model was employed to assess the association between RAR and 28-day mortality. Results were reported as hazard ratios (HRs) with 95% confidence intervals (CI). Three sequential models were constructed: Model I (unadjusted); Model II (adjusted for age, sex, ethnicity, and BMI); Model III (further adjusted for comorbidities, laboratory parameters, vital signs, and disease severity scores, including mechanical ventilation, creatinine, lactate, APACHE IV score, and SOFA score). Handling of missing data: Variables with a missing rate exceeding 30% were excluded from the analysis. The missing rates for the remaining variables were all below 10%. Multiple Interpolation of Chained Equations (MICE) was performed using the “mice” package in R, generating five interpolated datasets, each iterated 20 times, and the predicted mean matching method was applied to continuous variables. The interpolation models included all variables from Model III. Details of missing variables are provided in Supplementary Table S2A. As a sensitivity analysis, we also conducted a complete-cases analysis (excluding patients with missing data for any covariates), and the results were similar to those of the analysis described above (Supplementary Table S2B). To assess the potential survival bias arising from the exclusion of patients with an intensive care unit (ICU) stay of ≤ 24 h, we included patients who died within 24 h and conducted a sensitivity analysis (Supplementary Table S2C). Assessment of multicollinearity: To assess multicollinearity, the variance inflation factors (VIFs) were calculated for all variables in Model III. VIF > 5 was considered problematic.

The proportional hazards assumption was verified using Schoenfeld residuals. Kaplan-Meier survival curves were plotted for the two RAR groups, and survival distributions were compared using the log-rank test (19). Subgroup analyses were conducted according to age, sex, and key comorbidities, with interaction terms tested (20). To explore potential nonlinear relationships between continuous RAR and mortality risk, four-knot constrained cubic splines (RCS) were fitted within fully adjusted Cox models. Following Harrell's recommendation, the node positions were set at the 5th, 35th, 65th and 95th percentiles; an AIC comparison confirmed that the 4-node model was the most favorable. Two-tailed *P* values < 0.05 were considered statistically significant. All analyses were performed using R statistical software (version 4.2.2; R Statistical Computing Foundation) and SPSS (version 26.0; IBM Corporation).

## 3 Result

### 3.1 Baseline characteristics of the study population

A total of 13,888 adult patients with sepsis were ultimately included. The median age was 67.0 years (interquartile range 56.0–78.0), with women accounting for 51.7%. Participants were stratified into a low RAR group (RAR < 5.9, *n* = 6,944) and a high RAR group (RAR ≥ 5.9, *n* = 6,944) based on a median RAR score of 5.9. Table 1 summarizes baseline demographic, clinical, and laboratory characteristics across both groups. Patients in the high RAR group were older, had a higher proportion of Caucasian ethnicity, and exhibited more severe clinical symptoms, with higher APACHE IV scores (77.0 ± 24.6 vs. 67.1 ± 22.5, *P* < 0.001) and SOFA scores (4.8 ± 2.8 vs. 4.0 ± 2.5, *P* < 0.001). serum albumin levels were lower (2.2 ± 0.5 vs. 3.2 ± 0.5 g/dl, *P* < 0.001), RDW values were higher (16.7% vs. 14.4%, *P* < 0.001), and organ dysfunction indicators such as creatinine, lactate, and blood urea nitrogen were more markedly abnormal (all *P* < 0.001). The ICU mortality rate (14.4% vs 6.7%, *P* < 0.001) and in-hospital mortality rate (23.0% vs 9.9%, *P* < 0.001) were significantly elevated in the high RAR group (Table 1).

**Table 1:** Baseline characteristics and outcome parameters of participants.

Variables	Total (*N* = 13,888)	Low RAR (*N* = 6,944)	High RAR (*N* = 6,944)	*P*-value
Age, (years)	67.0 (56.0, 78.0)	67.0 (55.0, 78.0)	68.0 (57.0, 78.0)	0.016
Gender, *n* (%)				
Female	7,179 (51.7%)	3,686 (53.1%)	3,493 (50.3%)	0.001
Male	6,709 (48.3%)	3,258 (46.9%)	3,451 (49.7%)	
BMI, (kg/m^2^)	28.1 ± 7.1	28.5 ± 7.1	27.7 ± 7.1	< 0.001
Race, *n* (%)				
African American	1,303 (9.4%)	559 (8.1%)	744 (10.7%)	< 0.001
Asian	275 (2%)	148 (2.1%)	127 (1.8%)	
Caucasian	10,996 (79.2%)	5,603 (80.7%)	5,393 (77.7%)	
Hispanic	519 (3.7%)	242 (3.5%)	277 (4%)	
Native American	679 (4.9%)	354 (5.1%)	325 (4.7%)	
Other	116 (0.8%)	38 (0.5%)	78 (1.1%)	
Hypertension, *n* (%)				
No	12,297 (88.5%)	6,032 (86.9%)	6,265 (90.2%)	< 0.001
Yes	1,591 (11.5%)	912 (13.1%)	679 (9.8%)	
Diabetes, *n* (%)				
No	13,381 (96.3%)	6,716 (96.7%)	6,665 (96%)	0.024
Yes	507 (3.7%)	228 (3.3%)	279 (4%)	
Pneumonia, *n* (%)				
No	9,594 (69.1%)	4,654 (67%)	4,940 (71.1%)	< 0.001
Yes	4,294 (30.9%)	2,290 (33%)	2,004 (28.9%)	
Chronic renal insufficiency, *n* (%)				
No	13,038 (93.9%)	6,537 (94.1%)	6,501 (93.6%)	0.215
Yes	850 (6.1%)	407 (5.9%)	443 (6.4%)	
Myocardial infarction, *n* (%)				
No	13,334 (96%)	6,613 (95.2%)	6,721 (96.8%)	< 0.001
Yes	554 (4%)	331 (4.8%)	223 (3.2%)	
Arrhythmia, *n* (%)				
No	11,610 (83.6%)	5,820 (83.8%)	5,790 (83.4%)	0.506
Yes	2,278 (16.4%)	1,124 (16.2%)	1,154 (16.6%)	
Heart failure, *n* (%)				
No	12,494 (90%)	6,181 (89%)	6,313 (90.9%)	< 0.001
Yes	1,394 (10%)	763 (11%)	631 (9.1%)	
End stage renal disease, *n* (%)				
No	13,288 (95.7%)	6,695 (96.4%)	6,593 (94.9%)	< 0.001
Yes	600 (4.3%)	249 (3.6%)	351 (5.1%)	
Chronic obstructive pulmonary disease, *n* (%)				
No	12,611 (90.8%)	6,222 (89.6%)	6,389 (92%)	< 0.001
Yes	1,277 (9.2%)	722 (10.4%)	555 (8%)	
Mechanical ventilation, *n* (%)				
No	9,601 (69.1%)	5,053 (72.8%)	4,548 (65.5%)	< 0.001
Yes	4,287 (30.9%)	1,891 (27.2%)	2,396 (34.5%)	
RDW/ALB, (%/g/dl)	5.9 (4.8, 7.4)	4.8 (4.2, 5.3)	7.4 (6.5, 8.9)	< 0.001
RDW, (%)	15.4 (14.2, 17.1)	14.4 (13.6, 15.5)	16.7 (15.2, 18.6)	< 0.001
RBC, ( × 10^12^/L)	3.6 (3.1, 4.2)	3.9 (3.4, 4.3)	3.4 (2.9, 3.9)	< 0.001
WBC, ( × 10^9^/L)	13.6 (9.4, 19.1)	13.4 (9.6, 18.1)	13.9 (9.0, 20.1)	0.007
PLT, ( × 10^9^/L)	193.0 (133.0, 267.0)	194.5 (144.5, 258.5)	190.0 (118.0, 278.0)	< 0.001
Hemoglobin, (%)	10.8 ± 2.2	11.7 ± 2.1	10.0 ± 1.9	< 0.001
Total bilirubin, (mg/dl)	0.7 (0.4, 1.2)	0.7 (0.4, 1.1)	0.8 (0.4, 1.3)	< 0.001
Lymph, (%)	9.5 (5.0, 10.5)	9.5 (5.1, 11.5)	9.8 (5.0, 10.1)	< 0.001
Lac, (mmol/L)	2.5 (1.5, 2.7)	2.4 (1.5, 2.6)	2.5 (1.5, 2.8)	0.005
Cr, (mg/dl)	1.4 (0.9, 2.3)	1.3 (0.9, 2.1)	1.5 (0.9, 2.5)	< 0.001
BUN, (mg/dl)	28.0 (17.0, 45.0)	25.0 (16.0, 40.5)	31.0 (18.7, 49.5)	< 0.001
Ph	7.3 (7.3, 7.4)	7.3 (7.3, 7.4)	7.3 (7.3, 7.4)	0.096
ALB, (g/dl)	2.7 ± 0.7	3.2 ± 0.5	2.2 ± 0.5	< 0.001
K, (mmol/L)	4.1 ± 0.7	4.1 ± 0.7	4.1 ± 0.7	0.385
Na, (mmol/L)	137.8 ± 7.1	137.6 ± 7.3	138.0 ± 6.9	< 0.001
Temperature, (°C)	35.9 ± 6.1	36.2 ± 5.4	35.6 ± 6.8	< 0.001
Heartrate, (bpm)	93.1 ± 16.5	91.9 ± 16.1	94.2 ± 16.8	< 0.001
respiratory rate, (bpm)	21.4 ± 4.7	21.4 ± 4.7	21.4 ± 4.8	0.793
Systolic blood pressure, (mmHg)	112.0 ± 16.5	114.9 ± 17.5	109.2 ± 15.1	< 0.001
Diastolic blood pressure, (mmHg)	61.6 ± 10.3	63.1 ± 10.7	60.1 ± 9.6	< 0.001
Apache score	72.0 ± 24.1	67.1 ± 22.5	77.0 ± 24.6	< 0.001
Sofa score	4.4 ± 2.7	4.0 ± 2.5	4.8 ± 2.8	< 0.001
ICU mortality rate, *n* (%)				
No	12,421 (89.4%)	6,480 (93.3%)	5,941 (85.6%)	< 0.001
Yes	1,467 (10.6%)	464 (6.7%)	1,003 (14.4%)	
Hosp mortality rate, *n* (%)				
No	11,603 (83.5%)	6,254 (90.1%)	5,349 (77%)	< 0.001
Yes	2,285 (16.5%)	690 (9.9%)	1,595 (23%)	

Continuous variables are expressed as mean ± standard deviation (SD) for normally distributed data or median (interquartile range, IQR) for non-normally distributed data. Categorical variables are presented as number (percentage, %). *P* < 0.05 indicates statistical significance. RAR: Red blood cell distribution width to albumin ratio; BMI: Body mass index; RDW: Red blood cell distribution width; RBC: Red blood cell count; WBC: White blood cell count; PLT: Platelet count; Lymph: Lymphocyte percentage; Lac: Lactate; Cr: Creatinine; BUN: Blood urea nitrogen; ALB: Albumin; K: Potassium; Na: Sodium; SOFA: Sequential Organ Failure Assessment score.

### 3.2 Multivariate Cox regression analysis of RAR and mortality rate

The results of the Cox proportional hazards regression analysis indicated that, in the unadjusted model (Model I), a high RAR (median group) was significantly associated with an increased risk of ICU mortality (HR 1.83, 95% CI 1.64–2.04, *P* < 0.001) and hospital mortality (HR 1.87, 95% CI 1.71–2.04, *P* < 0.001). When RAR was treated as a continuous variable, a one-unit increase was associated with an 11% increase in the risk of ICU mortality (HR 1.11, 95% CI 1.10–1.13, *P* < 0.001) and an 11% increase in the risk of in-hospital mortality (HR 1.11, 95% CI 1.10–1.12, *P* < 0.001). These associations remained statistically significant after continuous adjustment for potential confounders. In the fully adjusted model (Model III), which included demographic characteristics, comorbidities, laboratory parameters, vital signs and disease severity scores, a high RAR (median grouping) was associated with an increased risk of ICU mortality (HR 1.34, 95% CI 1.19–1.50, *P* < 0.001) and increased in-hospital mortality (HR 1.50, 95% CI 1.36–1.64, *P* < 0.001). Similarly, when RAR was analyzed as a continuous variable, a 1-unit increase in RAR was associated with a 8% increase in ICU mortality (HR 1.08, 95% CI 1.06–1.09, *P* < 0.001) and an 9% increase in in-hospital mortality (HR 1.08, 95% CI 1.07–1.10, *P* < 0.001), representing increases of 8% and 9% respectively (Table 2). To further elucidate the dose-response relationship, we employed quartile analysis as a sensitivity analysis method (Supplementary Table S4). The results were consistent with the binary analysis: compared with the lowest quartile (Q1), the fully adjusted HR for ICU mortality was 1.21 (95% CI 1.00–1.47) in Q2, 1.26 (1.05–1.52) in Q3, and 1.70 (1.42–2.03) in Q4 (trend *P*-value < 0.001); for in-hospital mortality, the corresponding HRs were 1.17 (1.00–1.37), 1.36 (1.17–1.58) and 1.88 (1.63–2.17) (trend *P*-value < 0.001). Multicollinearity diagnosis (Supplementary Table S3): None of the VIF values for the variables in Model III exceeded 5, indicating that there is no significant multicollinearity.

**Table 2:** Univariate and multivariate Cox regression analyses for the association between RAR and mortality outcomes.

	Model 1		Model 2		Model 3	
RDW/ALB ration	HR (95%CI)	*P*–value	HR (95%CI)	*P*–value	HR (95%CI)	*P*–value
ICU mortality						
Low RAR	Ref		Ref		Ref	
High RAR	1.83 (1.64–2.04)	*p* < 0.001	1.78 (1.59–1.99)	*p* < 0.001	1.34 (1.19–1.50)	*p* < 0.001
Continuous variable	1.11 (1.10–1.13)	*p* < 0.001	1.11 (1.10–1.13)	*p* < 0.001	1.08 (1.06–1.09)	*p* < 0.001
Hospital mortality						
Low RAR	Ref		Ref		Ref	
High RAR	1.87 (1.71–2.04)	*p* < 0.001	1.83 (1.68–2.01)	*p* < 0.001	1.50 (1.36–1.64)	*p* < 0.001
Continuous variable	1.11 (1.10–1.12)	*p* < 0.001	1.12 (1.10–1.13)	*p* < 0.001	1.09 (1.07–1.10)	*p* < 0.001

Model I: Unadjusted. Model II: Adjusted for gender, age, race, and body mass index (BMI). Model III: Adjusted for variables in Model II plus hypertension, diabetes, pneumonia, chronic renal insufficiency, myocardial infarction, arrhythmia, heart failure, end-stage renal disease, chronic obstructive pulmonary disease (COPD), mechanical ventilation, creatinine, lactate, blood urea nitrogen (BUN), potassium (K), sodium (Na), temperature, heart rate, systolic blood pressure, diastolic blood pressure, APACHE IV score, GCS score, and SOFA score. RAR: Red blood cell distribution width to albumin ratio; HR: Hazard ratio; CI: Confidence interval.

### 3.3 Survival analysis

**Figure 2:**
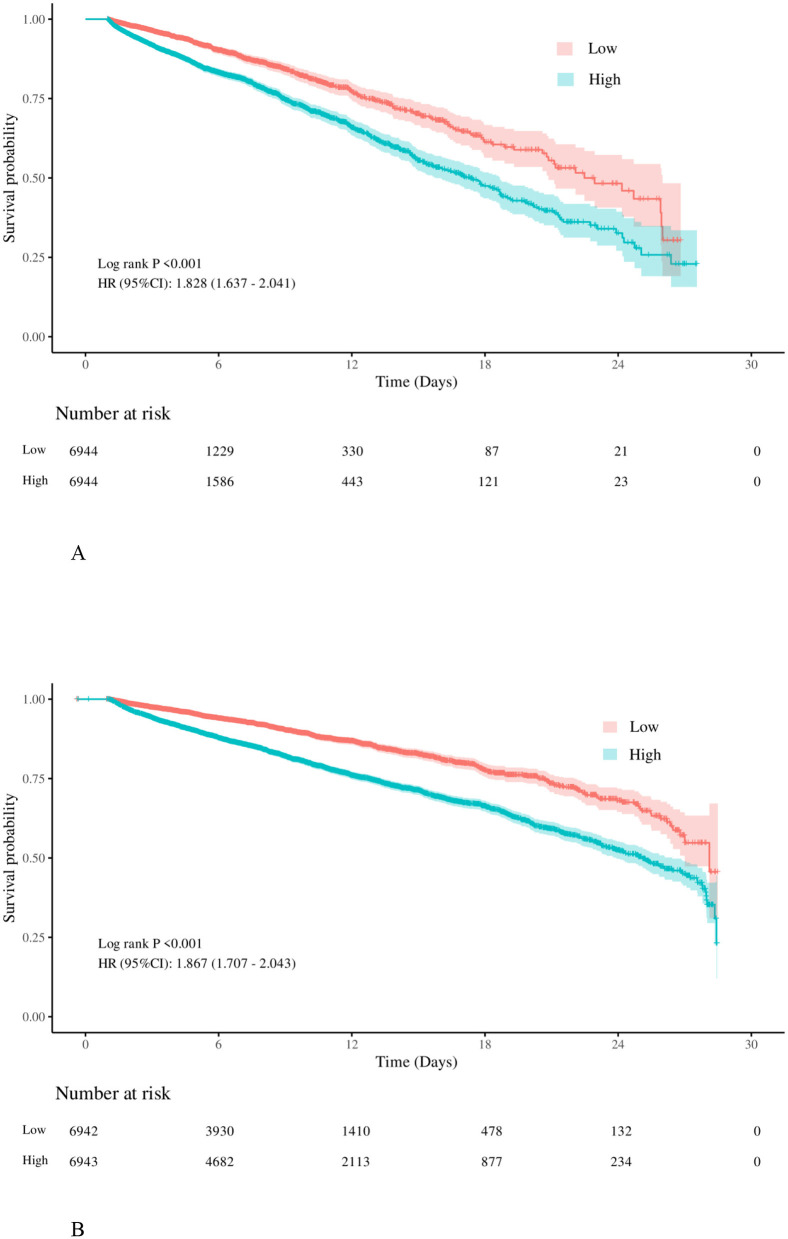
**(A)** Kaplan–Meier survival curves for patients with sepsis stratified by RAR level during their first 28 days in the ICU. **(B)** Kaplan–Meier survival curves for patients with sepsis stratified by RAR level during their first 28 days of hospitalization.

Kaplan-Meier survival curves demonstrated significant differences in ICU survival rates (log-rank *P* < 0.001, Figure 2A) and hospital survival rates (log-rank *P* < 0.001, Figure 2B) between the low RAR and high RAR groups. Throughout the 28-day follow-up period, patients in the high RAR group consistently exhibited poorer survival probabilities.

### 3.4 Subgroup analyses

**Figure 3:**
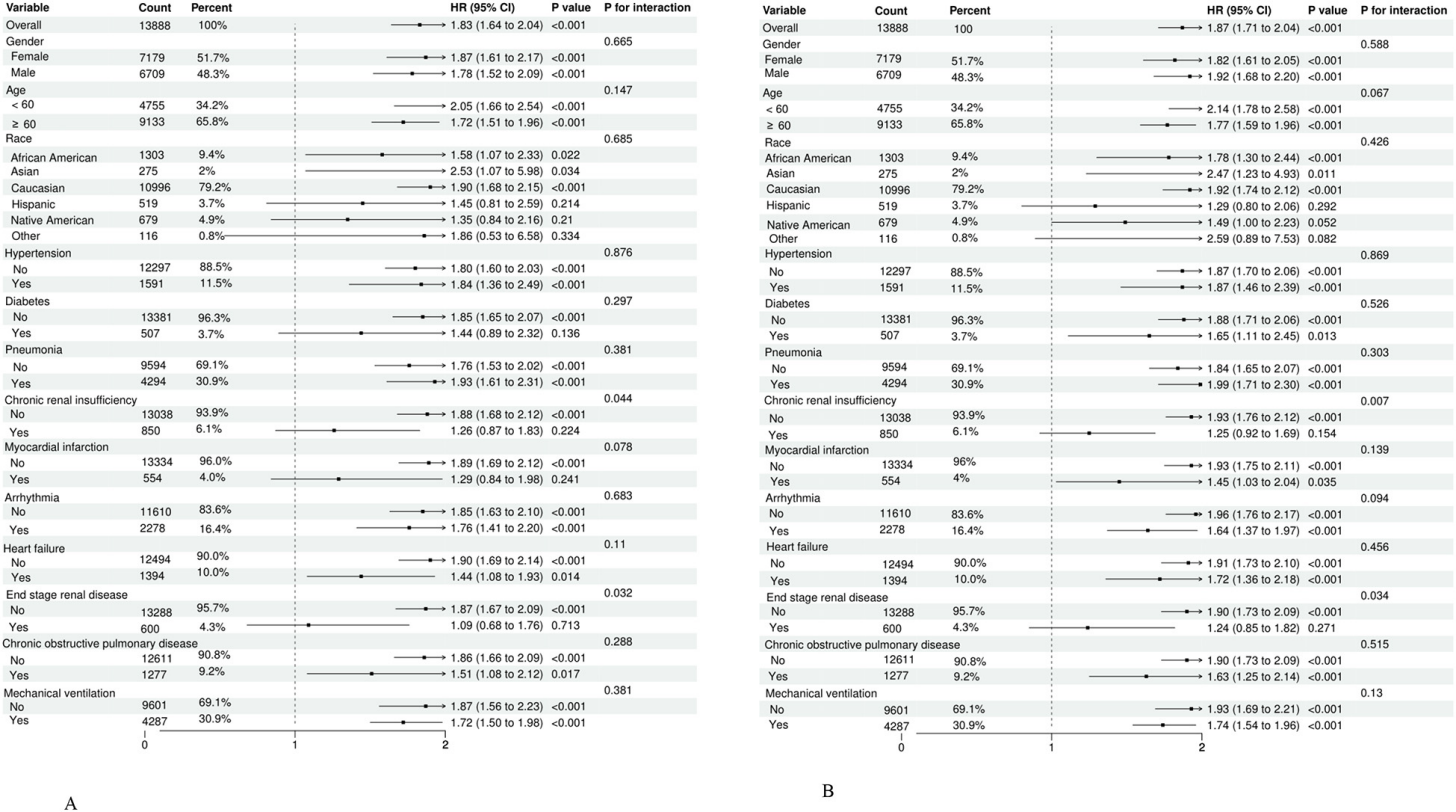
**(A)** Subgroup analysis forest plot of RAR and ICU mortality rates. **(B)** Subgroup analysis forest plot of RAR versus in-hospital mortality.

Subgroup analyses were conducted to assess the consistency of the association between high RAR (≥ median) and mortality across different patient strata (Figure 3). Across all pre-specified subgroups, both ICU mortality (Figure 3A) and in-hospital (28-day) mortality (Figure 3B) consistently demonstrated an increased risk associated with high RAR. These included stratification by age (< 60 years vs. ≥60 years), sex, and the presence of key comorbidities such as hypertension, diabetes mellitus, and chronic kidney disease. No significant interactions for either outcome were detected in any subgroup (all interactions *P* > 0.05), indicating that the prognostic value of elevated RAR is robust across these distinct patient cohorts.

### 3.5 Nonlinear relationship analysis

**Figure 4:**
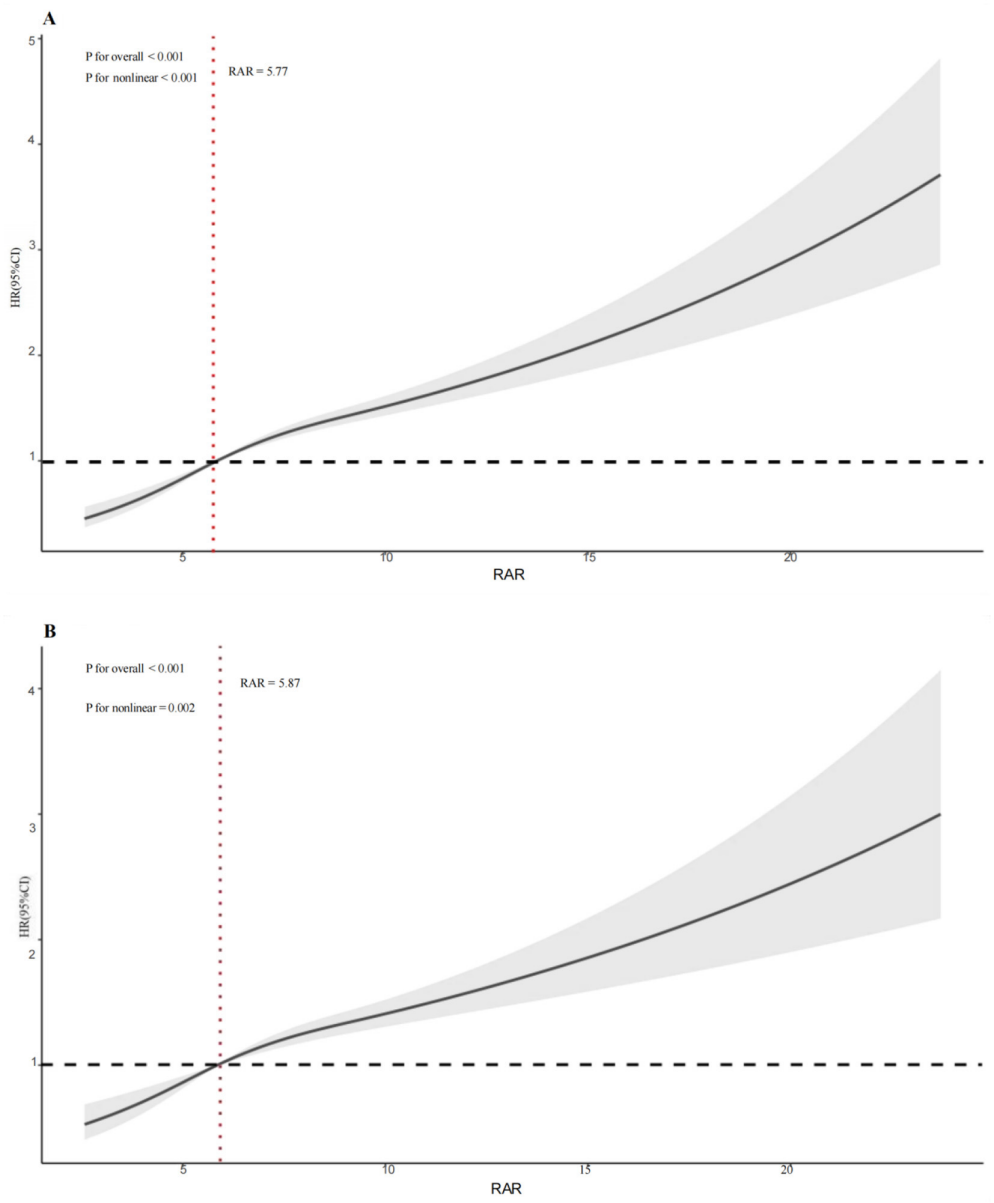
Nonlinear dose-response relationship between RAR and mortality outcomes. **(A)** In-hospital mortality. **(B)** ICU mortality.

Restricted cubic spline analysis revealed a non-linear dose-response relationship between continuous RAR and mortality risk. For in-hospital mortality (Figure 4A), risk increased gradually with rising RAR values, followed by a marked steep rise beyond an RAR threshold of approximately 5.77, indicating a threshold effect. For ICU mortality (Figure 4B), a similar non-linear pattern was evident, with mortality risk increasing more rapidly above the RAR threshold (approximately 5.87). Both the overall and non-linear associations for these outcomes were statistically significant (both *P* < 0.01), indicating that the relationship between RAR and mortality is non-linear, with risk accelerating markedly once RAR exceeds these critical levels. To assess whether the thresholds were affected by overfitting, we conducted tertile and quintile analyses (Supplementary Tables S5 and S6). In the tertile analysis, compared with the lowest tertile (T1), the hazard ratio (HR) for intensive care unit mortality in the T3 group was 1.82 (95% CI 1.57–2.11); the HR for in-hospital mortality was 1.90 (95% CI 1.68–2.13). In the quintile analysis, compared with the lowest quintile (Q1), the HR for ICU mortality in the highest quintile (Q5) was 2.16 (1.76–2.64), and the HR for in-hospital mortality was 2.27 (1.93–2.67). These results were consistent with the quartile and median analyses, ruling out the possibility of overfitting.

## 4 Discussion

In this large, multicentre retrospective cohort study utilizing the eICU CRD database, we found that an elevated red blood cell distribution width-to-albumin ratio (RAR) upon admission to the intensive care unit (ICU) was independently and significantly associated with increased all-cause mortality within 28 days. After extensive adjustment for demographics, comorbidities, laboratory parameters, and validated disease severity scores, elevated RAR remained a significant predictor of adverse outcomes. Our findings further reveal consistent prognostic value across key patient subgroups and identify a non-linear, dose-dependent relationship between RAR and mortality risk, with the increase becoming more pronounced beyond a threshold value.

Comparison with previous eICU-based studies. Two previous eICU-based studies have examined the RAR in elderly patients with sepsis: Hu and Qian (15) reported a potential non-linear association but did not use RCS for formal validation; whereas An et al. (16) reported a linear relationship using a generalized additive model. In contrast, the present study possesses the following methodological advantages: (1) it included adult sepsis patients across all age groups, providing a broader study population; (2) it formally tested for non-linear relationships using RCS combined with strict threshold identification (verified via the bootstrap method); (3) We conducted interaction tests to confirm consistency across subgroups; (4) We performed multiple sensitivity analyses (tertilisation, quintilisation, quartilisation, and complete-case analysis) to rule out overfitting and selection bias.

Clinical significance beyond established severity scores. Although the SOFA and APACHE IV scores are well-validated predictors of mortality in sepsis, they require multiple clinical and laboratory parameters, which are often difficult to calculate immediately at the bedside in resource-limited settings. In contrast, RAR is based solely on two routine and low-cost laboratory tests. Whilst RAR should not replace these composite scores, as an easily accessible adjunctive marker, it may offer additional prognostic value. In our fully adjusted model, RAR remained independently associated with 28-day mortality even after adjusting for SOFA and APACHE IV scores (Table 2, Model III), suggesting that RAR captures additional risk information beyond these established severity indices. However, future studies require direct comparisons of predictive performance (e.g., AUC, NRI) to quantify the incremental value of RAR.

Our findings align with the majority of evidence supporting the prognostic role of composite biomarkers integrating inflammation and nutritional status in critical illness (9). Previous studies have confirmed the independent prognostic value of elevated RDW and hypoalbuminaemia in sepsis (21–23). RDW reflects underlying inflammation, oxidative stress, and impaired erythropoietic function-core components of sepsis pathophysiology (24, 25). Conversely, albumin is a negative acute-phase reactant whose levels decline due to systemic inflammation, capillary leakage, and synthetic dysfunction (25–27). By combining these two inversely correlated markers (9, 28), RAR potentially amplifies their individual prognostic signals, thereby more comprehensively reflecting the severity of the host dysregulation response (29, 30). Whilst prior studies have linked RAR to prognosis in conditions such as severe pneumonia and septic shock (12, 31), our research specifically confirms RAR's strong association with the clinically critical 28-day mortality endpoint across a broad septic population.

The intertwined pathophysiological pathways represented by RAR support the biological plausibility of our findings. Sepsis-induced systemic inflammation directly suppresses bone marrow function and disrupts iron metabolism, leading to the release of immature erythrocytes and increased RDW (32–34). Concurrently, pro-inflammatory cytokines promote endothelial dysfunction and capillary leakage, resulting in albumin extravasation and catabolism (28, 35). Consequently, elevated RAR may signify a composite state of biological injury: persistent inflammatory haematopoietic dysregulation accompanied by marked protein-energy malnutrition and diminished plasma osmotic pressure (9, 24). This synergistic effect may exacerbate microcirculatory dysfunction, impair tissue oxygenation, worsen organ failure, and ultimately contribute to increased mortality (12, 19).

It is noteworthy that our subgroup analyses revealed no significant interactions, indicating that the association between elevated RAR and increased mortality risk persists consistently across age, sex, and the presence of common comorbidities such as hypertension or diabetes. This robustness strengthens the argument for RAR as a broadly applicable risk stratification tool. Furthermore, restricted cubic spline analysis revealed a non-linear relationship, indicating that mortality risk does not increase linearly but accelerates beyond a certain RAR threshold (approximately >5.87 in our cohort). This “threshold effect” implies that a critical point may exist where the combined deterioration of erythropoietic homeostasis and nutritional integration status accelerates patient prognosis decline beyond a certain threshold. Identifying such a threshold could prove valuable for defining high-risk populations in clinical practice.

From a clinical perspective, the main advantages of the RAR lie in its simplicity and cost-effectiveness. Red blood cell distribution width (RDW) and albumin are both routine tests that are readily available and inexpensive. No additional resources are required to calculate this ratio. However, the findings of this study are still at an exploratory stage and should not be overinterpreted as evidence supporting immediate clinical application. As a routine prognostic tool, its clinical utility requires validation through prospective cost-effectiveness studies before it can be recommended for widespread use, particularly in resource-limited settings (7, 28).

Our study has several limitations worthy of consideration. Firstly, as a retrospective observational study, its design inherently precludes causal inferences, despite our comprehensive multivariate adjustments. Unmeasured confounding factors and selection bias cannot be entirely ruled out; therefore, the observed associations should be interpreted as suggestive rather than conclusive. Secondly, to ensure a comprehensive assessment of exposure, we excluded patients with an ICU stay of ≤ 24 h (*n* = 1,847, of whom 182 died, yielding a mortality rate of 9.85%). This exclusion may have introduced survival bias. However, sensitivity analyses including these patients yielded consistent results, indicating that our main findings did not change substantially. Nevertheless, as the mortality rate among the excluded patients was lower than that of the included cohort (9.85% vs. 16.5%), this exclusion may have slightly attenuated the observed effect size. Thirdly, we used only the first RAR value obtained within 24 h of admission to the ICU and did not assess its dynamic changes over time (e.g., on day 3 or day 7). This is a significant limitation, as sepsis is a dynamic process and a single-point-in-time measurement may fail to capture changes in the host response. Continuous measurement of the RAR may provide additional prognostic information or reflect treatment response, but this remains to be explored in future prospective studies. Fourthly, the generalisability of our findings to non-US populations or to septic patients outside the ICU setting requires further validation. Fifth, although we adjusted for a broad range of covariates, residual confounding from factors not captured in the database—such as detailed microbiological data, specific treatment regimens, or pre-hospital functional status-remains possible. Finally, these limitations underscore the fact that our findings should be regarded as preliminary and speculative in nature, and require validation in well-designed prospective cohort studies.

Future studies should utilize independent databases (such as MIMIC-IV) to conduct external validation, in order to confirm the generalisability of the findings of this study across different ICU settings and patient populations. Furthermore, future research could develop a simple clinical prediction model that combines RAR with existing severity scores (such as SOFA or APACHE IV) to improve bedside risk stratification. Such tools could provide personalized assessments of the probability of death and aid clinical decision-making. Furthermore, mechanistic studies are required to further elucidate the causal relationship between elevated RAR and poor outcomes in sepsis.

## 5 Conclusion

This large-scale, multicentre study confirms that elevated RAR levels upon ICU admission constitute an independent predictor of increased 28-day mortality in septic patients. This association persists after extensive adjustment for confounding factors, remains consistent across key subgroups, and exhibits a non-linear dose-response relationship. As a simple, cost-effective and readily available indicator, RAR may serve as a potential adjunct for early risk stratification. Given the exploratory and retrospective nature of this study, these findings should be regarded as hypothesis-generating. Before definitive recommendations can be made, its clinical value across different healthcare settings needs to be confirmed through prospective studies.

## Data Availability

The datasets presented in this study can be found in online repositories. The names of the repository/repositories and accession number(s) can be found below: https://eicu-crd.mit.edu.
