# Supplementary material for: The prognostic value of red cell distribution width-to-albumin ratio for 28-day mortality in sepsis patients: a multicenter analysis based on the eICU Collaborative Research Database
Source: Front Med (Lausanne). 2026 May 28;13:1816709. doi: 10.3389/fmed.2026.1816709 (PMC13253725; doi:10.3389/fmed.2026.1816709)
Supplement: Supplementary file 1 [file Table_1.docx]

**Supplementary Table S1**

| **Study (First author, year)** | **Database/Data source** | **Sample size** | **Population restriction** | **Primary outcome(s)** | **RAR grouping method** | **Nonlinear analysis (RCS)** | **Collinearity diagnosis (VIF)** | **Sensitivity analysis for bias exclusion** | **Other methodological highlights** |
| --- | --- | --- | --- | --- | --- | --- | --- | --- | --- |
| Xu et al., 2022 | MIMIC‑IV | 14,639 | Adult sepsis | 28-day, 90-day, in-hospital mortality | Tertiles | Not performed | Not reported | Not reported | First large-sample MIMIC study |
| Huang et al., 2024 | MIMIC‑III | 1,475 | Adult sepsis | 28-day mortality | Unspecified (Continuous) | Not performed | Not reported | Not reported | Developed prediction model |
| Gu et al., 2022 | MIMIC‑IV | 3,042 | Sepsis + Atrial fibrillation | In-hospital mortality | Continuous | Linear (No RCS) | Not reported | Not reported | Restricted to AF population |
| Yao et al., 2025 | MIMIC‑IV | 4,021 | Sepsis-associated delirium | 30-day mortality | Binary (Optimal cutoff 5.85) | Not performed | Not reported | PSM | Focused on delirium subgroup |
| Wu et al., 2026 | MIMIC‑IV | 6,042 | Sepsis-associated ARDS | 30, 90, 180, 365-day mortality | Continuous + Quartiles | RCS (Nonlinear, inflection 5.04) | Not reported | PSM | Focused on ARDS subgroup |
| Li et al., 2025 | MIMIC‑IV | 2,100 | Sepsis + Cirrhosis | 30, 365-day mortality | Quartiles | RCS (Linear) | Not reported | Not reported | Focused on cirrhosis subgroup |
| Yang et al., 2025 | MIMIC‑IV | 1,686 | Sepsis + Malignancy | 28-day in-hospital mortality | Quartiles | RCS (Linear) | Not reported | Not reported | Focused on malignancy subgroup |
| Hu et al., 2025 | eICU‑CRD | 17,321 | Elderly sepsis | 28-day mortality | Tertiles | Mentioned "possible nonlinearity" | Not reported | Not reported | First eICU study in elderly |
| An et al., 2025 | eICU‑CRD | 5,976 | Elderly (≥60 yrs) sepsis | 28-day in-hospital mortality | Quartiles | GAM (Linear) | Not reported | Not reported | Focused on elderly |
| Present study (Ye et al., 2026) | eICU‑CRD | 13,888 | All adults (≥18 yrs) sepsis | 28-day ICU mortality + In-hospital mortality | Median groups+ Continuous | RCS Nonlinear (Threshold 5.87) | Complete VIF report (APACHE IV 2.56, etc.) + Model sensitivity analysis | Exclusion of ICU stay ≤24h + Pre-transfusion sampling timestamp clarification | Subgroup interaction tests (Age/Sex/Comorbidities) |

**Supplementary Table S2A**

| Missing variables | Percentage of missing data (N=13888) |
| --- | --- |
| BMI | 728 (5.24%) |
| RBC | 31(0.22%) |
| WBC | 18(0.13%) |
| PLT | 58(0.42%) |
| Lac | 292(2.11%) |
| Cr | 27(0.19%) |
| Bun | 19(0.14%) |
| K | 16(0.12%) |
| Na | 11(0.08%) |
| Ph | 581(4.18%) |
| Temperature | 388(2.79%) |
| Heartrate | 885(6.37%) |
| Apache score | 303(2.18%) |
| GCS | 316(2.27%) |
| SOFA | 294(2.11%) |

**Supplementary Table S2B**

|  | Modle1 | Modle2 | Modle3 |
| --- | --- | --- | --- |
| RDW/ALB ration | HR(95%CI) P-value | HR(95%CI) P-value | HR(95%CI) P-value |
| **ICU mortality** |  | | |
| Low RAR | Ref | Ref | Ref |
| High RAR | 1.74 (1.56-1.94) <0.001 | 1.73 (1.55-1.93) <0.001 | 1.47 (1.31-1.65) <0.001 |
| continuous variable | 1.10 (1.09-1.12) <0.001 | 1.11 (1.09-1.12) <0.001 | 1.08 (1.06-1.09) <0.001 |
| **Hospital mortality** | | | |
| Low RAR | Ref | Ref | Ref |
| High RAR | 1.74 (1.59-1.90) <0.001 | 1.75 (1.60-1.91) <0.001 | 1.56 (1.43-1.71) <0.001 |
| continuous variable | 1.10 (1.09-1.12) <0.001 | 1.11 (1.10-1.12) <0.001 | 1.09 (1.07-1.10) <0.001 |

Notes:
Model I: Unadjusted.
Model II: Adjusted for gender, age, race, and body mass index (BMI).
Model III: Adjusted for variables in Model II plus hypertension, diabetes, pneumonia, chronic renal insufficiency, myocardial infarction, arrhythmia, heart failure, end-stage renal disease, chronic obstructive pulmonary disease (COPD), mechanical ventilation, creatinine, lactate, blood urea nitrogen (BUN), potassium (K), sodium (Na), temperature, heart rate, systolic blood pressure, diastolic blood pressure, APACHE IV score, GCS score, and SOFA score.
RAR: Red blood cell distribution width to albumin ratio; HR: Hazard ratio; CI: Confidence interval.

**Supplementary Table 2C**

|  | Modle1 | Modle2 | Modle3 |
| --- | --- | --- | --- |
| RDW/ALB ration | HR(95%CI) P-value | HR(95%CI) P-value | HR(95%CI) P-value |
| **ICU mortality** |  | | |
| Low RAR | Ref | Ref | Ref |
| High RAR | 1.71 (1.55-1.90) <0.001 | 1.69 (1.53-1.88) <0.001 | 1.36 (1.22-1.51) <0.001 |
| continuous variable | 1.10 (1.09-1.12) <0.001 | 1.10 (1.09-1.12) <0.001 | 1.07 (1.05-1.08) <0.001 |
| **Hospital mortality** | | | |
| Low RAR | Ref | Ref | Ref |
| High RAR | 1.74 (1.60-1.89) <0.001 | 1.74 (1.60-1.89) <0.001 | 1.48 (1.36-1.62) <0.001 |
| continuous variable | 1.10 (1.09-1.11) <0.001 | 1.11 (1.10-1.12) <0.001 | 1.08 (1.07-1.09) <0.001 |

Notes:
Model I: Unadjusted.
Model II: Adjusted for gender, age, race, and body mass index (BMI).
Model III: Adjusted for variables in Model II plus hypertension, diabetes, pneumonia, chronic renal insufficiency, myocardial infarction, arrhythmia, heart failure, end-stage renal disease, chronic obstructive pulmonary disease (COPD), mechanical ventilation, creatinine, lactate, blood urea nitrogen (BUN), potassium (K), sodium (Na), temperature, heart rate, systolic blood pressure, diastolic blood pressure, APACHE IV score, GCS score, and SOFA score.
RAR: Red blood cell distribution width to albumin ratio; HR: Hazard ratio; CI: Confidence interval.

**Supplementary Table S3**

| Variables | VIF |
| --- | --- |
| SOFA | 2.22 |
| APACHE IV | 2.56 |
| GCS | 1.98 |
| Age | 1.42 |
| Gender | 1.06 |
| BMI | 1.09 |
| Race | 1.02 |
| Hypertension | 1.13 |
| Diabetes | 1.07 |
| Pneumonia | 1.08 |
| Chronic renal insufficiency | 1.05 |
| Myocardial infarction | 1.02 |
| Arrhythmia | 1.10 |
| Heart failure | 1.13 |
| End stage renal disease | 1.36 |
| Chronic obstructive pulmonary disease | 1.06 |
| Mechanical ventilation | 1.43 |
| Lac | 1.23 |
| Cr | 2.35 |
| Bun | 2.09 |
| K | 1.23 |
| Na | 1.21 |
| Temperature | 1.19 |
| Heartrate | 1.37 |
| Systolic blood pressure | 1.89 |
| Diastolic blood pressure | 1.99 |

**Supplementary Table S4**

|  | Modle1 | Modle2 | Modle3 |
| --- | --- | --- | --- |
|  | HR(95%CI) P-value | HR(95%CI) P-value | HR(95%CI) P-value |
| **ICU mortality** |  | | |
| RAR Quartiles |  |  |  |
| Q1 | Ref | Ref | Ref |
| Q2 | 1.54 (1.27-1.86) <0.001 | 1.46 (1.21-1.76) <0.001 | 1.21 (1.00-1.47) 0.048 |
| Q3 | 1.83 (1.53-2.19) <0.001 | 1.73 (1.44-2.07) <0.001 | 1.26 (1.05-1.52) 0.014 |
| Q4 | 2.82 (2.38-3.34,) <0.001 | 2.68 (2.26-3.18) <0.001 | 1.70 (1.42-2.03) <0.001 |
| Trend for P | < 0.001 | < 0.001 | < 0.001 |
| **Hospital mortality** | | | |
| RAR Quartiles |  |  |  |
| Q1 | Ref | Ref | Ref |
| Q2 | 1.44 (1.23-1.68) <0.001 | 1.36 (1.17-1.59) <0.001 | 1.17 (1.00-1.37) 0.043 |
| Q3 | 1.84 (1.59-2.13) <0.001 | 1.74 (1.50-2.01) <0.001 | 1.36 (1.17-1.58) <0.001 |
| Q4 | 2.74 (2.39-3.15) <0.001 | 2.65 (2.30-3.04) <0.001 | 1.88 (1.63-2.17) <0.001 |
| Trend for P | < 0.001 | < 0.001 | < 0.001 |

Notes:
Model I: Unadjusted.
Model II: Adjusted for gender, age, race, and body mass index (BMI).
Model III: Adjusted for variables in Model II plus hypertension, diabetes, pneumonia, chronic renal insufficiency, myocardial infarction, arrhythmia, heart failure, end-stage renal disease, chronic obstructive pulmonary disease (COPD), mechanical ventilation, creatinine, lactate, blood urea nitrogen (BUN), potassium (K), sodium (Na), temperature, heart rate, systolic blood pressure, diastolic blood pressure, APACHE IV score, GCS score, and SOFA score.
RAR: Red blood cell distribution width to albumin ratio; HR: Hazard ratio; CI: Confidence interval.

**Supplementary Table S5**

|  | Modle1 | Modle2 | Modle3 |
| --- | --- | --- | --- |
|  | HR(95%CI) P-value | HR(95%CI) P-value | HR(95%CI) P-value |
| **ICU mortality** |  | | |
| RAR tertiles |  |  |  |
| T1 | Ref | Ref | Ref |
| T2 | 1.55 (1.33-1.81) <0.001 | 1.49 (1.27-1.74) <0.001 | 1.25 (1.07-1.47) 0.005 |
| T3 | 2.50 (2.17-2.89) <0.001 | 2.45 (2.13-2.83) <0.001 | 1.82 (1.57-2.11) <0.001 |
| P for trend | < 0.001 | < 0.001 | < 0.001 |
| **Hospital mortality** | | | |
| RAR tertiles |  |  |  |
| T1 | Ref | Ref | Ref |
| T2 | 1.44 (1.27-1.64) <0.001 | 1.38 (1.21-1.56) <0.001 | 1.20 (1.06-1.36) 0.005 |
| T3 | 2.40 (2.14-2.69) <0.001 | 2.37 (2.11-2.65) <0.001 | 1.90 (1.68-2.13) <0.001 |
| P for trend | < 0.001 | < 0.001 | < 0.001 |

Notes:
Model I: Unadjusted.
Model II: Adjusted for gender, age, race, and body mass index (BMI).
Model III: Adjusted for variables in Model II plus hypertension, diabetes, pneumonia, chronic renal insufficiency, myocardial infarction, arrhythmia, heart failure, end-stage renal disease, chronic obstructive pulmonary disease (COPD), mechanical ventilation, creatinine, lactate, blood urea nitrogen (BUN), potassium (K), sodium (Na), temperature, heart rate, systolic blood pressure, diastolic blood pressure, APACHE IV score, GCS score, and SOFA score.
RAR: Red blood cell distribution width to albumin ratio; HR: Hazard ratio; CI: Confidence interval.

**Supplementary Table S6**

|  | Modle1 | Modle2 | Modle3 |
| --- | --- | --- | --- |
|  | HR(95%CI) P-value | HR(95%CI) P-value | HR(95%CI) P-value |
| **ICU mortality** |  | | |
| RAR Quartiles |  |  |  |
| Q1 | Ref | Ref | Ref |
| Q2 | 1.51 (1.21-1.88) <0.001 | 1.48 (1.18-1.84) <0.001 | 1.32 (1.06-1.65) 0.014 |
| Q3 | 1.66 (1.34-2.06) <0.001 | 1.56 (1.26-1.94) <0.001 | 1.28 (1.02-1.59) 0.030 |
| Q4 | 2.21 (1.79-2.71) <0.001 | 2.12 (1.72-2.61) <0.001 | 1.65 (1.33-2.03) <0.001 |
| Q5 | 3.22 (2.65-3.92) <0.001 | 3.13 (2.57-3.81) <0.001 | 2.16 (1.76-2.64) 0.014 |
| P for trend | < 0.001 | < 0.001 | < 0.001 |
| **Hospital mortality** | | | |
| RAR Quartiles |  |  |  |
| Q1 | Ref | Ref | Ref |
| Q2 | 1.46 (1.22-1.74) <0.001 | 1.41 (1.18-1.69) <0.001 | 1.29 (1.08-1.54) 0.006 |
| Q3 | 1.64 (1.37-1.95) <0.001 | 1.53 (1.28-1.82) <0.001 | 1.27 (1.06-1.51) 0.008 |
| Q4 | 2.19 (1.85-2.58) <0.001 | 2.11 (1.78-2.49) <0.001 | 1.73 (1.46-2.04) <0.001 |
| Q5 | 3.08 (2.62-3.61) <0.001 | 3.02 (2.57-3.54) <0.001 | 2.27 (1.93-2.67) <0.001 |
| P for trend | < 0.001 | < 0.001 | < 0.001 |

Notes:
Model I: Unadjusted.
Model II: Adjusted for gender, age, race, and body mass index (BMI).
Model III: Adjusted for variables in Model II plus hypertension, diabetes, pneumonia, chronic renal insufficiency, myocardial infarction, arrhythmia, heart failure, end-stage renal disease, chronic obstructive pulmonary disease (COPD), mechanical ventilation, creatinine, lactate, blood urea nitrogen (BUN), potassium (K), sodium (Na), temperature, heart rate, systolic blood pressure, diastolic blood pressure, APACHE IV score, GCS score, and SOFA score.
RAR: Red blood cell distribution width to albumin ratio; HR: Hazard ratio; CI: Confidence interval.
